# Efficacy of local budesonide therapy in the management of persistent hyposmia in COVID-19 patients without signs of severity: A structured summary of a study protocol for a randomised controlled trial

**DOI:** 10.1186/s13063-020-04585-8

**Published:** 2020-07-20

**Authors:** Mary Daval, Alain Corré, Clement Palpacuer, Juliette Housset, Guillaume Poillon, Michael Eliezer, Benjamin Verillaud, Dorsaf Slama, Denis Ayache, Philippe Herman, Clement Jourdaine, Camille Hervé, Wissame El Bakkouri, Dominique Salmon, Charlotte Hautefort

**Affiliations:** 1 Hopital Fondation Adolphe de Rothschild, Paris, France; 2 Hopital Lariboisiere, Paris, France; 3 Hotel Dieu Hospital, Paris, France

**Keywords:** COVID-19, Randomised controlled trial, rotocol, hyposmia, dysgeusia, nasal irrigation, corticosteroid, budesonide, MRI

## Abstract

**Objectives:**

To assess the efficacy of local intranasal treatment with budesonide (nasal irrigation), in addition to olfactory rehabilitation, in the management of loss of smell in COVID-19 patients without signs of severity and with persistent hyposmia 30 days after the onset of symptoms.

To search for an association between the presence of an obstruction on MRI and the severity of olfactory loss, at inclusion and after 30 days of treatment.

**Trial design:**

Two center, open-label, 2-arm (1:1 ratio) parallel group randomized controlled superiority trial.

**Participants:**

**Inclusion criteria**

- Patient over 18 years of age;

- Patient with a suspected SARS-CoV-2 infection, whether or not confirmed by PCR, or close contact with a PCR-confirmed case, typical chest CT scan (unsystematic frosted glass patches with predominantly sub-pleural appearance, and at a later stage, alveolar condensation without excavation or nodules or masses) or positive serology ;

- Patient with isolated sudden onset hyposmia persisting 30 days after the onset of symptoms of CoV-2 SARS infection;

- Affiliate or beneficiary of a social security scheme;

- Written consent to participate in the study.

**Non-inclusion criteria**

- Known hypersensitivity to budesonide or any of the excipients;

- Hemostasis disorder or epistaxis;

- Oral-nasal and ophthalmic herpes virus infection;

- Long-term corticosteroid treatment;

- Treatment with potent CYP3A4 inhibitors (e.g., ketoconazole, itraconazole, voriconazole, posaconazole, clarithromycin, telithromycin, nefazodone and HIV protease inhibitors);

- Severe forms of SARS-CoV-2 with respiratory or other signs;

- Hyposmia persisting for more than 90 days after the onset of symptoms

- Other causes of hyposmia found on interrogation or MRI;

- Patient benefiting from a legal protection measure;

- Pregnant or breastfeeding women.

The participants will be recruited from: Hôpital Fondation Adolphe de Rothschild and Hôpital Lariboisière in Paris, France

**Intervention and comparator:**

Intervention:

**Experimental group:** Nasal irrigation with budesonide and physiological saline (Budesonide 1mg/2mL diluted in 250mL of physiological saline 9°/00): 3 syringes of 20mL in each nasal cavity, morning and evening, for 30 days, in addition to olfactory rehabilitation twice a day.

**Control group:** Nasal irrigation with physiological saline 9°/00 only: 3 syringes of 20cc in each nasal cavity, morning and evening, for 30 days, in addition to olfactory rehabilitation twice a day.

**Main outcomes:**

Percentage of patients with an improvement of more than 2 points on the ODORATEST score after 30 days of treatment.

**Randomisation:**

Patients will be randomized (1:1) between the experimental and control groups, using the e-CRF. The randomization list will be stratified by centre.

**Blinding (masking):**

Participants and caregivers are aware of the group assignment. People assessing the outcomes are blinded to the group assignment

Numbers to be randomised (sample size)

120 patients are planned to be randomized into two groups of 60 patients.

**Trial Status:**

MDL_2020_10. Version number 2, May 22, 2020. Recruitment started on May 22, 2020. The trial will finish recruiting by August 2020.

**Trial registration:**

**EUDRACT number:** 2020-001667-85; date of trial registration: 15 May 2020

Protocol registered on ClinicalTrial.gov, registration number: NCT04361474; date of trial registration: 24 April 2020.

**Full protocol:**

The full protocol is attached as an additional file, accessible from the Trials website (Additional file 1). In the interest in expediting dissemination of this material, the familiar formatting has been eliminated; this Letter serves as a summary of the key elements of the full protocol.

## Supplementary information

**Additional file 1.** Full Study Protocol.

## Data Availability

Each investigator will have access to the final trial dataset. Project data sets are housed on the Project Accept Web site (Clinfile), and all data sets are password protected. Project Principal Investigators have direct access to their own site’s data sets. To ensure confidentiality, data dispersed to project team members will be blinded of any identifying participant information data will be available from the author on reasonable request (mdaval@for.paris).

