# Supplementary material for: Efficacy of local budesonide therapy in the management of persistent hyposmia in COVID-19 patients without signs of severity: A structured summary of a study protocol for a randomised controlled trial
Source: Trials. 2020 Jul 20;21:666. doi: 10.1186/s13063-020-04585-8 (PMC7370627; doi:10.1186/s13063-020-04585-8)
Supplement: Supplementary file 1 — Additional file 1. Full Study Protocol. [file 13063_2020_4585_MOESM1_ESM.pdf]

HÔPITAL FONDATION
Adolphe de ROTHSCHILD
LA RÉFÉRENCE TÊTE ET COU

PROTOCOLE DE RECHERCHE INTERVENTIONNELLE
PORTANT SUR UN MÉDICAMENT

COVIDORL

Essai randomisé contrôlé évaluant l'efficacité d'un traitement local par budésonide dans la prise en charge de l'hyposmie chez les patients COVID19 sans signes de gravité.

Code de la recherche : MDL_2020_10

N° EUDRACT : 2020-001667-85

Version du protocole n° 2 du 25/05/2020

Avis favorable du CPP Sud-Est II le 29/04/2020

Autorisation de l'ANSM le 14/05/2020

**Promoteur**	**Hôpital Fondation Adolphe de Rothschild**<br>29 rue Manin<br>75019 Paris<br>**Représentant du promoteur :**<br>Dr Amélie Yavchitz, cheffe de service adjointe<br>Service de Recherche Clinique / DRCI<br>Tél : 01 48 03 64 54 Fax : 01 48 03 64 30<br>Mail : ayavchitz@for.paris

**Investigateur coordonnateur**	Dr Mary DAVAL<br>Service d'ORL<br>Hôpital Fondation Adolphe de Rothschild<br>Tél : 01 48 03 68 39<br>Mail : mdaval@for.paris

**Investigateur associé**	Dr Charlotte Hautefort<br>Service ORL<br>Hôpital Lariboisière<br>2 rue Ambroise Paré<br>75010 Paris<br>Mail : charlotte.hautefort@aphp.fr

Ce document confidentiel est la propriété de la Fondation Adolphe de Rothschild. Aucune information non publiée figurant dans ce document ne peut être divulguée sans autorisation écrite préalable de la Fondation Adolphe de Rothschild.

PAGE DE SIGNATURE DU PROTOCOLE

<u>Titre et acronyme de l'étude :</u> Essai randomisé contrôlé évaluant l'efficacité d'un traitement local par budésonide dans la prise en charge de l'hyposmie chez les patients COVID-19 sans signes de gravité (COVIDORL).

Code de la recherche : MDL_2020_10

Version du protocole n° 2 du 25/05/2020

Ce protocole a été lu et approuvé à la date notée ci-dessous.

Les deux parties s'engagent à mener la recherche conformément au protocole, aux bonnes pratiques cliniques, et aux dispositions législatives et réglementaires en vigueur.

POUR LE PROMOTEUR :

Dr Amélie Yavchitz, cheffe de service adjointe
Service de Recherche Clinique / DRCI
Hôpital Fondation Adolphe de Rothschild

Date : 25/05/2020
Signature :

L'INVESTIGATEUR COORDONNATEUR :

Dr Mary DAVAL
Service d'ORL
Hôpital Fondation Adolphe de Rothschild

Date : 25/05/2020
Signature :

TABLE DES MATIERES

LISTE DES ABRÉVIATIONS **5**
SYNOPSIS **6**
1 Introduction - justification scientifique **12**
2 Objectifs de la recherche **14**
2.1 Objectif principal 14
2.2 Objectifs secondaires 14
3 Critères de jugement **14**
3.1 Critère de jugement principal 14
3.2 Critères de jugement secondaires 14
4 Sélection et exclusion des personnes de la recherche **15**
4.1 Population cible et mode de recrutement 15
4.2 Critères d'inclusion 15
4.3 Critères de non inclusion 15
5 Description de l'étude **16**
5.1 Type d'étude 16
5.2 Déroulement de la recherche 17
5.2.1 Centres investigateurs 17
5.2.2 Modalités de prise en charge des patients 17
5.2.3 Modalités de sorties d'étude 19
5.2.4 Recueil des données 20
5.2.5 Tableau récapitulatif du déroulement de l'étude 21
5.3 Calendrier de l'étude 22
5.4 Bénéfices, risques et contraintes pour le patient participant à la recherche 22
6 Description des médicaments de l'étude **23**
6.1 Identification du traitement 23
6.2 Conditionnement et étiquetage 23
6.3 Commande et distribution des traitements 23
6.4 Administration 23
6.5 Adaptation de la posologie 23
6.6 Traitements associés, autorisés, non autorisés et de secours dans le cadre du protocole 24
7 Evaluation de la sécurité **24**
7.1 Définitions 24
7.2 Enregistrement et notification des évènements indésirables par l'investigateur 25
7.2.1 Evènements indésirables 25
7.2.2 Evènements indésirables graves 25
7.2.3 Evènement indésirables graves ne nécessitant pas de notification immédiate 26
7.3 Prise en charge des évènements indésirables graves par le promoteur 26
7.3.1 Evaluation médicale 26
7.3.2 Déclaration des suspicions d'effets indésirables graves inattendus (SUSAR) 27
7.4 Fait nouveau et mesures urgentes de sécurité 27
7.5 Rapport annuel de sécurité 27
7.6 Comité de surveillance indépendant 27
8 Analyses statistiques **28**
8.1 Calcul du nombre de sujets 28
8.2 Description des analyses statistiques utilisées 28
8.2.1 Choix des personnes à inclure dans les analyses 28
8.2.2 Statistiques descriptives 28
8.2.3 Comparaison des groupes à la baseline 28
8.2.4 Analyse du critère de jugement principal 28
8.2.5 Analyse des critères de jugement secondaires 28
8.3 Niveau de significativité statistique 30

8.4 Critères statistiques d’arrêt de la recherche 30
8.5 Modalités de prise en compte des données manquantes, non utilisées ou non valides 30
8.6 Gestion des modifications apportées au plan statistique initial 30
9 Dispositions réglementaires 30
9.1 Demande d’autorisation auprès de l’autorité compétente (Agence Nationale de Sécurité du Médicament et des produits de santé - ANSM) 30
9.2 Demande d’avis auprès du Comité de Protection des Personnes (CPP) 30
9.3 Engagements – aspects éthiques 30
9.4 Déclaration du démarrage et de la fin de l’étude 31
9.5 Note d’information et recueil du consentement écrit du patient 31
9.6 Modifications du protocole 31
9.7 Déclaration CNIL (Commission Nationale de l’Informatique et des Libertés) 31
9.8 Droits d’accès aux données et documents sources 32
9.9 Archivage 32
10 Assurance et financement 33
10.1 Assurance 33
10.2 Financement 33
11 Règles relatives à la publication et rapport final 33
11.1 Enregistrement de l’étude 33
11.2 Rapport final 33
11.3 Communication et publication des résultats 33
12 Références 35
13 Annexes 36
13.1 Annexe 1. Test olfactif ODORATEST 36
13.2 Annexe 2. Questionnaire d’auto-évaluation 37
13.3 Annexe 3 : Protocole de rééducation olfactive 39

LISTE DES ABRÉVIATIONS

AMM : Autorisation de mise sur le marché

ANSM : Agence Nationale de Sécurité du Médicament et des produits de santé

CNIL : Commission Nationale de l'Informatique et des Libertés

CPP : Comité de Protection des Personnes

CSI : Comité de Surveillance Indépendant

CT: Computerized tomography

COVID-19 : Maladie à coronavirus 2019

e-CRF : Electronic Case Report Form

EI : Evénement Indésirable

EIG : Événement Indésirable Grave

IRM : Imagerie par Résonnance Magnétique

LFN : Lavages des fosses nasales

OMS Organisation Mondiale de la Santé

ORL : Oto-rhino-laryngologie

RGPD : Règlement Général sur la Protection des Données de l'Union européenne

SARS-Cov-2: Severe acute respiratory syndrome coronavirus 2

SUSAR : Suspected Unexpected Serious Adverse Reaction

SYNOPSIS

**Titre de la recherche**	Essai randomisé contrôlé évaluant l'efficacité d'un traitement local par budésonide dans la prise en charge de l'hyposmie chez les patients COVID-19 sans signes de gravité.
**Acronyme**	**COVIDORL**
**Promoteur**	Hôpital Fondation Adolphe de Rothschild<br>29 rue Manin<br>75019 Paris
**Investigateur coordonnateur**	Dr Mary Daval<br>Service d'ORL<br>Hôpital Fondation Adolphe de Rothschild
**Nombre de centres**	2
**Justification de la recherche**	Les symptômes initiaux décrits chez les premiers cas de COVID-19 étaient essentiellement la fièvre et les signes respiratoires. Récemment, des médecins ont constaté dans plusieurs pays (1) une augmentation des cas de perte de l'odorat, sans obstruction nasale ni rhinorrhée associée. Même si nous ne connaissons pas encore les conséquences au long cours du COVID-19 sur les capacités olfactives, il existe des données de la littérature confirmant que les hyposmies post-virales sont une source importante de troubles olfactifs sur le long terme, impactant la qualité de vie (2).<br>Habituellement, le traitement des hyposmies virales repose sur un traitement corticoïde par voie locale et/ou générale associé à des lavages de nez au sérum physiologique dès l'apparition des signes. La société française d'ORL a, du fait de la possible gravité de l'atteinte virale à SARS-Cov-2, déconseillé le traitement par corticothérapie ainsi que les lavages de nez associés à une rééducation olfactive. Cependant, le virus étant en moyenne présent dans les fosses nasales pendant 20 jours (3), une hyposmie persistante à 30 jours serait probablement due à une atteinte inflammatoire ou neurologique des fentes ou bulbe olfactif (objectivée ou non par l'IRM). Un traitement local par corticoïdes pourrait être instauré à partir de 30 jours du début des symptômes de COVID-19 sans risque de dissémination.<br>Dans les hyposmies persistantes hors rhinosinusites chroniques, le seul traitement (4) ayant prouvé son efficacité comporte des lavages de nez associés au budésonide et à une rééducation olfactive (5). Ce médicament n'a toutefois pas l'AMM en France dans cette indication.<br>Un essai thérapeutique testant un traitement associant des lavages de nez comportant du budésonide à une rééducation olfactive chez les patients présentant une hyposmie persistante à 30 jours du début des signes d'infection à SARS-Cov-2 parait justifié. L'analyse des IRM pré et post thérapeutiques permettrait d'étudier également une éventuelle association entre une atteinte inflammatoire ou neurologique de l'organe de l'olfaction et la sévérité de la perte d'odorat.
**Objectif et critère de jugement principal**	**Objectif principal:** Evaluer l'efficacité du budésonide en traitement local intranasal (lavage de nez), en complément de la rééducation olfactive, dans la prise en charge de la perte d'odorat de patients COVID-19 sans signes de gravité et présentant une persistance de l'hyposmie 30 jours après le début des symptômes.

	**Critère de jugement principal:** Pourcentage de patients ayant une amélioration de plus de 2 points sur le score ODORATEST (5) après 30 jours de traitement.
**Objectifs et critères de jugement secondaires**	**Objectifs secondaires**:<br>1- Chez les patients ayant totalement récupéré (réponse de type oui/non à la question « Avez-vous totalement récupéré les capacités olfactives que vous aviez avant le début de l'apparition des symptômes de COVID-19 ? ») après 30 jours de traitement, évaluer la persistance de la récupération olfactive 1 mois après l'arrêt du traitement.<br>2- Dans le groupe traité par budésonide, chez les patients COVID-19 n'ayant pas totalement récupéré après 30 jours de traitement, évaluer l'intérêt d'une poursuite du budésonide pendant 30 jours supplémentaires.<br>3- Dans le groupe contrôle, chez les patients COVID-19 n'ayant pas totalement récupéré après 30 jours de traitement, évaluer l'intérêt de la mise en place d'un traitement par budésonide pendant 30 jours.<br>4- Rechercher une association entre la présence d'une obstruction à l'IRM et la sévérité de la perte olfactive, lors de l'inclusion et après 30 jours de traitement.<br>5- Evaluer la tolérance du budénoside.<br>6- Décrire les capacités olfactives et gustatives des patients à l'aide d'un questionnaire d'auto-évaluation.<br>7- Comparer au bout de 30 jours de traitement les capacités olfactives entre les deux bras.<br>8- Comparer au bout de 30 jours de traitement les capacités olfactives de type « détection des odeurs » entre les deux bras.<br>9- Comparer au bout de 30 jours de traitement les capacités olfactives de type « identification des odeurs » entre les deux bras.<br><br>**Critères de jugement secondaires:**<br>1- Pourcentage de patients ayant une amélioration de plus de 2 points au score ODORATEST 30 jours après la fin du traitement (i.e. 60 jours après la randomisation).<br>2- Pourcentage de patients ayant une amélioration de plus de 2 points au score ODORATEST après 60 jours de traitement par budésonide.<br>3- Pourcentage de patients ayant une amélioration de plus de 2 points au score ODORATEST 30 jours après l'initiation d'un traitement par budésonide (i.e. 60 jours après la randomisation).<br>4- Présence d'un comblement inflammatoire de la fente olfactive à l'IRM ou de la présence d'une atteinte neurologique au niveau du bulbe olfactif.<br>5- Description des évènements indésirables et événements indésirables graves.<br>6- Questionnaire d'auto-évaluation.<br>7- Score global ODORATEST après 30 jours de traitement<br>8- Sous-score détection de l'ODORATEST après 30 jours de traitement<br>9- Sous-score identification de l'ODORATEST après 30 jours

	de traitement
**Plan expérimental**	**Essai randomisé contrôlé, en ouvert, multicentrique.**
**Traitements à l'essai**	**<u>Groupe expérimental :</u>** Traitement par budésonide local intranasal mélangé à du sérum physiologique (Budésonide 1mg/2mL dilué dans 250mL de sérum physiologique 9°/00) en lavage de nez, 3 seringues de 20cc dans chaque narine matin et soir pendant 30 jours en complément de la rééducation olfactive (annexe 3) deux fois par jour.<br><br>**<u>Groupe contrôle :</u>** Traitement par lavages de nez, 3 seringues de 20cc de sérum physiologique 9°/00 dans chaque narine matin et soir pendant 30 jours en complément de la rééducation olfactive deux fois par jour.
**Population concernée**	File active de patients présentant une hyposmie isolée en lien avec une infection à SARS-CoV-2 sans signes de gravité, ayant consulté en ORL ou en médecine générale.
**Critères d'éligibilité**	**<u>Critères d'inclusion</u>:**<br>- Patient âgé de plus de 18 ans ;<br>- Patient avec une infection à SARS-CoV-2 suspectée dans un contexte épidémique, confirmée ou non par PCR, ou contact proche d'un cas confirmé par PCR, scanner thoracique typique (plages de verre dépoli non systématisées à prédominance sous-pleurale, et à un stade plus tardif de condensation alvéolaire sans excavations ni nodules ni masses) ou sérologie positive ;<br>- Patient présentant une hyposmie brutale isolée persistant à J30 du début des signes de l'infection à SARS-CoV-2 ;<br>- Absence de portage SARS-CoV-2 confirmée par PCR au moment de l'inclusion<br>- Affilié ou bénéficiaire d'un régime de sécurité sociale ;<br>- Consentement écrit de participation à l'étude.<br>**<u>Critères de non inclusion</u>:**<br>- Hypersensibilité connue au budésonide ou à un des excipients du médicament ;<br>- Trouble de l'hémostase, ou épistaxis ;<br>- Infection oro-bucco-nasale et ophtalmique par herpès virus ;<br>- Traitement par corticoïdes au long cours ;<br>- Traitement par inhibiteurs puissants du CYP3A4 (ex : kétoconazole, itraconazole, voriconazole, posaconazole, clarithromycine, télithromycine, néfazodone et inhibiteurs des protéases du VIH) ;<br>- Formes de SARS-CoV-2 avec signes respiratoires ou autres que l'anosmie persistant à 30 jours du début des symptômes ;<br>- Hyposmie persistante depuis plus de 90 jours après le début des symptômes ;<br>- Autres causes d'hyposmie mises en évidence à l'interrogatoire ou à l'IRM ;<br>- Patient bénéficiant d'une mesure de protection juridique ;<br>- Femme enceinte ou allaitante.<br><br>**Critères d'exclusion secondaire :**<br>Symptômes évocateurs d'une symptomatologie marquant une reprise

	de l'activité virale lors de l'appel téléphonique à J8.
**Déroulement pratique de l'essai**	✓ ***Screening :***<br>Les patients présentant une hyposmie brutale isolée persistant entre 30 et 90 jours après le début des symptômes d'une infection à SARS-CoV-2 suspectée par le tableau clinique et/ou confirmée par PCR sur écouvillon nasal ou ECBC, scanner thoracique ou sérologie seront contactés par téléphone afin de leur proposer de participer à l'étude et de vérifier les critères d'éligibilité.<br><br>Si les patients sont intéressés, la note d'information leur sera envoyée par mail et une prescription pour réaliser une PCR en ville. Une consultation physique dans un des deux centres inclueurs (Hôpital Fondation Adolphe de Rothschild, Hôpital Lariboisière) sera programmée entre J30 et J90 après le début des premiers symptômes d'infection à SARS-Cov-2.<br><br>✓ ***Inclusion (J-1):***<br>Au cours de la consultation, le médecin investigateur recueillera le consentement de participation à l'étude. Si le patient répond « non » à la question « Avez-vous totalement récupéré les capacités olfactives que vous aviez avant le début de l'apparition des symptômes de COVID-19 », il pourra être inclus dans l'étude. Un test olfactif sera réalisé. Il s'agit du test de perception et d'identification ODORATEST (5) de cinq odorants (rose, gâteau, fromage, abricot, fèces). Un questionnaire d'auto-évaluation des capacités olfactives et gustatives sera également réalisé. Un examen IRM 3T sera réalisé le même jour afin d'évaluer la présence de signes inflammatoires ou neurologiques. Il inclura en première intention des séquences non injectées couvrant l'encéphale (3D FLAIR, axiale diffusion, SWI) et les cavités naso-sinusiennes (Axial et Coronal 2D T2 et/ou 3D T2 HR). Une injection de produit de contraste pourra être requise secondairement, en cas de découverte d'une masse tumorale (méningiome olfactif, esthésioneuroblastome, tumeur sinusienne par exemple).<br><br>Après inclusion, les patients seront randomisés (1:1) entre le groupe expérimental et le groupe contrôle, à partir de l'e-CRF (Clinfile©). La liste de randomisation sera stratifiée sur le centre. Les patients se verront remettre les traitements à l'essai et auront pour instruction de démarrer le traitement à partir du lendemain matin.<br><br>✓ ***Traitement (J0-J29) :***<br>Groupe expérimental : traitement par budésonide local intranasal mélangé à du sérum physiologique (Budésonide 1mg/2mL dilué dans 250mL de sérum physiologique 9°/00) en lavage de nez, 3 seringues de 20cc dans chaque fosse nasale matin et soir pendant 30 jours en complément de la rééducation olfactive deux fois par jour.<br><br>Groupe contrôle : traitement par lavages de nez avec 3 seringues de 20cc de sérum physiologique 9°/00 dans chaque fosse nasale matin et soir pendant 30 jours en complément de la rééducation olfactive deux fois par jour.<br>✓ ***Appel téléphonique (J8) :*** vérification que le patient ne présente pas de réapparition de symptômes de COVID-19

	(fièvre, toux, gêne thoracique, dyspnée, asthénie…) et s'enquérir d'éventuelles difficultés liées au protocole de reconstitution et de réalisation des lavages de nez.<br><br>✓ ***<u>Visite de suivi n°1 (J15) :</u>***<br>Un appel téléphonique sera réalisé après 15 jours de traitement afin d'évaluer l'observance, et d'interroger le patient sur la survenue d'éventuels événements indésirables.<br><br>✓ ***<u>Visite de suivi n°2 (J30) :</u>***<br>Les patients seront revus entre 30 et 35 jours après la première prise de traitement. Le test olfactif sera réalisé et la survenue d'événements indésirables sera recueillie dans l'e-CRF. Le questionnaire d'auto-évaluation des capacités olfactives et gustatives sera réalisé. Un examen IRM de contrôle sera réalisé, selon les mêmes modalités que celui fait lors de l'inclusion.<br>Pour les patients ayant récupéré totalement (réponse de type oui/non à la question « Avez-vous totalement récupéré les capacités olfactives que vous aviez avant le début de l'apparition des symptômes de COVID-19 ? ») dans les deux groupes, le traitement par lavage de nez sera stoppé, mais il leur sera demandé de poursuivre la rééducation olfactive, deux fois par jour, jusqu'à la troisième visite de suivi.<br>Pour les patients du groupe expérimental n'ayant pas totalement récupéré, le traitement par budésonide en lavage de nez en complément de la rééducation olfactive sera poursuivi pendant 30 jours supplémentaires.<br>Pour les patients du groupe contrôle n'ayant pas totalement récupéré, le traitement par budésonide en lavage de nez sera instauré, en complément de la rééducation olfactive pendant 30 jours.<br><br>✓ ***<u>Visite de suivi n°3 (J60) :</u>***<br>Les patients seront revus à J60 (jusqu'à J70). Le test olfactif sera réalisé et la survenue d'événements indésirables sera recueillie dans l'e-CRF. Au cours de cette consultation sera également réalisé le questionnaire d'auto-évaluation des capacités olfactives et gustatives. A la suite de cette visite, la prise en charge des patients se fera selon les modalités habituelles du médecin qui les suit.<br><br>✓ ***<u>Visite de suivi n°4 (M8) :</u>***<br>Une consultation téléphonique sera réalisée à 8 mois (± 2 semaines) après l'inclusion des patients. Au cours de cette consultation sera réalisé le questionnaire d'auto-évaluation des capacités olfactives et gustatives. La participation des patients prendra fin à l'issue de cette consultation.
**Nombre de patients**	Nous faisons l'hypothèse que le pourcentage de patients présentant une amélioration olfactive dans le groupe contrôle sera d'environ 50% après 30 jours.<br>Afin de mettre en évidence une différence de 25% entre les deux bras (50% versus 75% de patients présentant une amélioration olfactive dans le bras traité par budésonide), environ 60 patients par

	bras sont nécessaires, **soit un total de 120 participants** (formulation bilatérale, puissance=0,8, alpha=0,05).
**Méthode statistique**	L'analyse du critère de jugement principal sera réalisée à l'aide d'un modèle de régression logistique, ajusté sur le centre.
**Calendrier prévisionnel**	Durée de la période d'inclusion : 3 mois<br>Durée de participation de chaque sujet : 8 mois<br>Durée totale de l'étude : 11 mois
**Références**	1. *Sixty seconds on . . . anosmia.* **Iacobucci, G.** 24 Mar 2020, BMJ , p. 1202.<br>2. *The relationship of olfactory function and clinical traits in major depressive disorder.* **Wang F, Wu X, Gao J, Li Y, Zhu Y, Fang Y.** 16, Mar 2020, Behav Brain Res , p. 112594.<br>3. *Clinical course and risk factors for mortality of adult inpatients with COVID-19 in Wuhan, China: a retrospective cohort study.* **Zhou, F, et al., et al.** 10229, 28 Mar 2020, Lancet, Vol. 395, pp. 1054-1062.<br>4. **Nguyen, TP et Patel, ZM.** Budesonide irrigation with olfactory training improves outcomes compared with olfactory training alone in patients with olfactory loss. *Int Forum Allergy Rhinol.* Sep 2018, Vol. 8, 9, pp. 977-981<br>5. *A new clinical olfactory test to quantify olfactory deficiences.* **Eloit, C et Trotier, D.** 1994, Rhinology.

COVIDORL

1 Introduction - justification scientifique

Les symptômes initiaux décrits chez les premiers cas de COVID-19 étaient essentiellement la fièvre et les signes respiratoires (1). Récemment, les médecins ont constaté, en France mais également dans d'autres pays (2), une augmentation importante des cas de perte d'odorat, sans obstruction nasale ni rhinorrhée associée. Une étude en cours de publication réalisée en France sur des patients présentant des symptômes de COVID-19 sans signe de gravité ainsi qu'une perte de l'odorat a montré que les tests réalisés chez ces patients confirmaient dans la quasi-totalité des cas l'infection à coronavirus SARS-CoV-2 (*in press*). Trois mécanismes pourraient expliquer cette perte d'odorat : (i) une atteinte neurotrope, avec atteinte élective des nerfs olfactifs et des bulbes (3) ; (ii) une atteinte muqueuse inflammatoire de la région de la fente olfactive, sans atteinte nerveuse ; (iii) une combinaison des deux. Même si nous ne connaissons pas encore les conséquences au long cours du COVID-19 sur les capacités olfactives, il existe des données de la littérature confirmant que les hyposmies post virales sont une source importante de troubles olfactifs au long cours impactant la qualité de vie (4).

Habituellement, le traitement des hyposmies virales repose sur un traitement corticoïde par voie locale et/ou générale associé à des lavages de nez au sérum physiologique dès l'apparition des signes. La société française d'ORL a, du fait de la possible gravité de l'atteinte virale, déconseillé le traitement par corticothérapie ainsi que les lavages de nez (2) associés à une rééducation olfactive. En effet, le virus SARS-CoV-2 pourrait rester présent dans la muqueuse des fosses nasales pendant 12 (5) à 20 jours (6) en moyenne avec un maxima à 38 jours dans les formes sévères (5), et présent jusqu'au décès dans les formes létales (6). Il ne nous paraît donc pas raisonnable de traiter par corticoïdes, même locaux, des patients encore porteurs du virus (risque potentiel de contamination pulmonaire ou de blocage de la réaction inflammatoire permettant de lutter contre la dissémination, risque de contamination de l'entourage). Un traitement instauré chez des patients ayant développé des symptômes de COVID-19 sans signes de gravité, et présentant une hyposmie persistante 30 jours après le début des symptômes parait en revanche envisageable, sans risque de dissémination.

Une récente méta-analyse (7) sur le traitement des troubles de l'olfaction hors rhinosinusite chronique ne retrouve pas d'étude à fort niveau de preuve en faveur de l'efficacité des corticoïdes par voie générale ou local en spray. Une seule étude de haut niveau de preuve a montré un intérêt des lavages de nez associé au budésonide (8) (7). Cette molécule, qui n'a pas l'AMM en France dans cette indication, serait efficace à distance de l'apparition de l'hyposmie.

Un essai thérapeutique testant un traitement associant des lavages de nez comportant du budésonide à une rééducation olfactive chez les patients présentant une hyposmie persistante à 30 jours du début des signes d'infection à SARS-Cov-2 parait justifié.

L'olfaction sera évaluée avant et après traitement grâce à des tests validés au niveau national et facilement reproductibles. Le test le plus couramment employé dans la littérature internationale est le test UPSIT (9) qui ne fait pas appel à des concentrations différentes d'odorants, qui n'a jamais donné lieu à une validation en France et qui est produit aux Etats-Unis. Dans le contexte de pandémie limitant les échanges postaux avec l'internationale et devant la nécessité de débuter l'étude rapidement, nous privilégierons un test facilement réalisable, reproductible et fiable, déjà validé dans une publication internationale, appelé ODORATEST (10). L'analyse des IRM pré et post thérapeutiques permettrait d'étudier également une éventuelle association entre une atteinte inflammatoire ou neurologique de l'organe de l'olfaction et la sévérité de la perte d'odorat.

Résumé de l'intérêt et de l'originalité du projet

Le virus SARS-CoV-2 entraîne des hyposmies pour laquelle la communauté scientifique française et internationale ne recommande pas le traitement habituel (corticoïdes quelle que soit la voie d'administration).
Le virus étant en moyenne présent dans les fosses nasales pendant 20 jours, un traitement local par corticoïdes pourrait être instauré à partir de 30 jours du début des signes de COVID-19, sans risque de dissémination.
Le seul traitement qui a prouvé son efficacité dans les hyposmies persistantes est la rééducation olfactive associée au budésonide, qui n'a pas l'AMM en France dans cette indication.
L'objectif de cet essai randomisé contrôlé, bicentrique, est d'évaluer l'efficacité du budésonide en traitement local intranasal (lavage de nez), en complément de la rééducation olfactive, dans la prise en charge de la perte d'odorat de patients COVID-19 sans signes de gravité et présentant une persistance de l'hyposmie 30 jours après le début des symptômes.

2 Objectifs de la recherche

2.1 Objectif principal

Evaluer l'efficacité du budésonide en traitement local intranasal (lavage de nez), en complément de la rééducation olfactive, dans la prise en charge de la perte d'odorat de patients COVID-19 sans signes de gravité et présentant une persistance de l'hyposmie 30 jours après le début des symptômes.

2.2 Objectifs secondaires

1- Chez les patients ayant totalement récupéré (réponse de type oui/non à la question « Avez-vous totalement récupéré les capacités olfactives que vous aviez avant le début de l'apparition des symptômes de COVID-19 ? ») après 30 jours de traitement, évaluer la persistance de la récupération olfactive 1 mois après l'arrêt du traitement.

2- Dans le groupe traité par budésonide, chez les patients COVID-19 n'ayant pas totalement récupéré après 30 jours de traitement, évaluer l'intérêt d'une poursuite du budésonide pendant 30 jours supplémentaires.

3- Dans le groupe contrôle, chez les patients COVID-19 n'ayant pas totalement récupéré après 30 jours de traitement, évaluer l'intérêt de la mise en place d'un traitement par budésonide pendant 30 jours.

4- Rechercher une association entre la présence d'une obstruction à l'IRM et la sévérité de la perte olfactive, lors de l'inclusion et après 30 jours de traitement.

5- Evaluer la tolérance du budénoside.

6- Décrire les capacités olfactives et gustatives des patients à l'aide d'un questionnaire d'auto-évaluation.

7- Comparer au bout de 30 jours de traitement les capacités olfactives entre les deux bras

8- Comparer au bout de 30 jours de traitement les capacités olfactives de type « détection des odeurs » entre les deux bras

9- Comparer au bout de 30 jours de traitement les capacités olfactives de type « identification des odeurs » entre les deux bras

3 Critères de jugement

3.1 Critère de jugement principal

Pourcentage de patients ayant une amélioration de plus de 2 points sur le score ODORATEST (10) après 30 jours de traitement (cf. **Annexe 1**).

3.2 Critères de jugement secondaires

1- Pourcentage de patients ayant une amélioration de plus de 2 points au score ODORATEST 30 jours après la fin du traitement (i.e. 60 jours après la randomisation).

2- Pourcentage de patients ayant une amélioration de plus de 2 points au score ODORATEST après 60 jours de traitement par budésonide.

3- Pourcentage de patients ayant une amélioration de plus de 2 points au score ODORATEST 30 jours après l'initiation d'un traitement par budésonide (i.e. 60 jours après la randomisation).

4- Présence d'un comblement inflammatoire de la fente olfactive à l'IRM ou présence d'une atteinte neurologique au niveau du bulbe olfactif.

5- Description des évènements indésirables et événements indésirables graves.

6- Questionnaire d'auto-évaluation (cf. **Annexe 2**)

7- Score global ODORATEST après 30 jours de traitement

8- Sous-score détection de l'ODORATEST après 30 jours de traitement

9- Sous-score identification de l'ODORATEST après 30 jours de traitement

4 Sélection et exclusion des personnes de la recherche

4.1 Population cible et mode de recrutement

File active de patients présentant une hyposmie isolée en lien avec une infection à SARS-CoV-2 sans signes de gravité, ayant consulté en ORL ou en médecine générale.

Les patients présentant une hyposmie brutale isolée persistant entre 30 et 90 jours après le début des symptômes d'une infection à SARS-CoV-2 suspectée par le tableau clinique et/ou confirmée par PCR sur écouvillon nasal ou ECBC, scanner thoracique ou sérologie seront contactés par téléphone afin de leur proposer de participer à l'étude et de vérifier les critères d'éligibilité.

4.2 Critères d'inclusion

- Patient âgé de plus de 18 ans ;
- Patient avec une infection à SARS-CoV-2 suspectée dans un contexte épidémique, confirmée ou non par PCR, ou contact proche d'un cas confirmé par PCR, scanner thoracique typique (plages de verre dépoli non systématisées à prédominance sous-pleurale, et à un stade plus tardif de condensation alvéolaire sans excavations ni nodules ni masses) ou sérologie positive ;
- Absence de portage SARS-CoV-2 confirmée par PCR au moment de l'inclusion
- Patient présentant une hyposmie brutale isolée persistant à J30 du début des signes de l'infection à SARS-CoV-2 ;
- Affilié ou bénéficiaire d'un régime de sécurité sociale ;
- Consentement écrit de participation à l'étude.

4.3 Critères de non inclusion

- Hypersensibilité connue au budésonide ou à un des excipients du médicament ;
- Trouble de l'hémostase, ou épistaxis ;
- Infection oro-bucco-nasale et ophtalmique par herpès virus ;
- Traitement par corticoïdes au long cours ;
- Traitement par inhibiteurs puissants du CYP3A4 (ex : kétoconazole, itraconazole, voriconazole, posaconazole, clarithromycine, télithromycine, néfazodone et inhibiteurs des protéases du VIH) ;
- Contre -indication à la réalisation d'une IRM ;
- Formes de SARS-CoV-2 avec signes respiratoires ou autres que l'anosmie persistant à 30 jours du début des symptômes ;

- Hyposmie persistante depuis plus de 90 jours après le début des symptômes ;
- Patient bénéficiant d'une mesure de protection juridique ;
- Femme enceinte ou allaitante.

4.4 Critères d'exclusion secondaire

- Symptômes évocateurs d'une symptomatologie marquant une reprise de l'activité virale lors de l'appel téléphonique à J8.

5 Description de l'étude

5.1 Type d'étude

Essai randomisé contrôlé, en ouvert, multicentrique.

5.2 Déroulement de la recherche

5.2.1 *Centres investigateurs*

Centre	Investigateur principal
Hôpital Fondation Adolphe de Rothschild<br>29 rue Manin<br>75019 Paris	Dr Mary Daval<br>Service ORL
Hôpital Lariboisière<br>2 rue Ambroise Paré<br>75010 Paris	Dr Charlotte Hautefort<br>Service ORL

5.2.2 *Modalités de prise en charge des patients*

✓ ***Screening :***

Les patients présentant une hyposmie brutale isolée persistant entre 30 et 90 jours après le début des symptômes d'une infection à SARS-CoV-2 sans signes de gravité, suspectée par le tableau clinique et/ou confirmée par PCR sur écouvillon nasal ou examen cytobactériologique des crachats, scanner thoracique ou sérologie, seront contactés par téléphone afin de leur proposer de participer à l'étude et de vérifier les critères d'éligibilité.

Si les patients sont intéressés, la note d'information leur sera envoyée par mail et une prescription pour la réalisation d'une PCR en ville. Une consultation physique dans un des deux centres investigateurs (Hôpital Fondation Adolphe de Rothschild ou Hôpital Lariboisière) sera programmée entre J30 et J60 après le début des premiers symptômes d'infection à SARS-Cov-2.

✓ ***Inclusion (J-1):***

Au cours de la consultation, le médecin investigateur recueillera le consentement écrit de participation à l'étude. Si le patient répond « non » à la question « Avez-vous totalement récupéré les capacités olfactives que vous aviez avant le début de l'apparition des symptômes de COVID-19 », il pourra être inclus dans l'étude. Dans le cahier d'observation (CRF) seront recueillies les données suivantes :

- Age, sexe, poids, taille, IMC, profession
- Consommation tabagique, immunosuppression, HTA, allergie, diabète
- Autres antécédents médicaux
- Antécédents ORL
- Contact cas confirmé
- Date de début des 1ers signes
- Date de début de l'anosmie
- Apparition isolée, concomitante ou après les autres signes
- Description de l'anosmie : partielle, totale, parosmies, phantosmies,
- Autres symptômes rhinologiques
- Troubles du gout
- Autres signes de COVID-19 : fièvre, frissons, toux, céphalée, myalgies, diarrhée, dyspnée, conjonctivite, laryngite
- Traitement reçu pour l'anosmie
- Traitement reçu pour le COVID-19

- Antibiothérapie
- Récupération et délai
- RT-PCR COVID-19 faite : résultat positif, négatif et charge virale
- Scanner thoracique fait et si oui résultat

Un test olfactif sera réalisé. Il s'agit du test de perception et d'identification ODORATEST (10) de cinq odorants (rose, gâteau, fromage, abricot, fèces), disponible en **Annexe 1** du protocole. Un questionnaire d'auto-évaluation des capacités olfactives et gustatives sera également réalisé (cf. **Annexe 2**).
Une IRM 3T sera effectuée le même jour afin d'évaluer la présence de signes inflammatoires ou neurologiques, conformément à la prise en charge habituelle pour le bilan d'une hyposmie. L'examen inclura en première intention des séquences non injectées couvrant l'encéphale (3D FLAIR, axiale diffusion, SWI) et les cavités naso-sinusiennes (Axial et Coronal 2D T2 et/ou 3D T2 HR). Une injection de produit de contraste pourra être requise secondairement, en cas de découverte d'une masse tumorale (e.g. méningiome olfactif, esthésioneuroblastome, tumeur sinusienne), conformément à ce qui est réalisé en pratique courante.

Après inclusion, les patients seront randomisés (1:1) entre le groupe expérimental et le groupe contrôle, à partir de l'e-CRF (Clinfile©). La liste de randomisation sera stratifiée sur le centre. Les patients se verront remettre les traitements à l'essai ainsi que le matériel nécessaire (seringues) et auront pour instruction de démarrer le traitement à partir du lendemain matin. Un carnet sera remis au patient. Ce carnet contiendra la notice détaillant les modalités de reconstitution et de réalisation des lavages de nez, le protocole de réalisation de la rééducation olfactive, un tableau permettant de suivre quotidiennement l'observance du traitement, ainsi qu'un rappel des principaux effets indésirables pouvant survenir avec le budésonide et les coordonnées des personnes à contacter en cas de survenue d'événements indésirables ou pour toute autre question concernant l'étude.

- ✓ ***Traitement (J0-J29) :***

Groupe expérimental : traitement par budésonide local intranasal mélangé à du sérum physiologique (Budésonide 1mg/2mL dilué dans 250mL de sérum physiologique 9°/00) en lavage de nez, 3 seringues de 20cc dans chaque narine matin et soir pendant 30 jours en complément de la rééducation olfactive deux fois par jour.

Groupe contrôle : traitement par lavages de nez avec 3 seringues de 20cc de sérum physiologique 9°/00 dans chaque narine matin et soir pendant 30 jours en complément de la rééducation olfactive deux fois par jour.

Le protocole de rééducation olfactive est détaillé en annexe 3.

- ✓ ***Appel téléphonique (J8) :*** vérification que le patient ne présente pas de réapparition de symptômes de COVID-19 (fièvre, toux, gêne thoracique, dyspnée, asthénie…) et s'enquérir d'éventuelles difficultés liées au protocole de reconstitution et de réalisation des lavages de nez.

- ✓ ***Visite de suivi n°1 (J15) :***

Un appel téléphonique sera réalisé après 15 jours de traitement afin d'évaluer l'observance, et d'interroger le patient sur la survenue d'éventuels événements indésirables qui seront colligés dans l'e-CRF.

✓ ***<u>Visite de suivi n°2 (J30) :</u>***

Les patients seront revus entre 30 et 35 jours après la première prise de traitement. Le test olfactif (ODORATEST) sera réalisé et la survenue d'événements indésirables sera recueillie dans l'e-CRF. Le questionnaire d'auto-évaluation des capacités olfactives et gustatives sera fait (cf. **Annexe 2**).

Un examen IRM de contrôle sera réalisé, selon les mêmes modalités que celui réalisé lors de l'inclusion.

Pour les patients ayant récupéré totalement (réponse de type oui/non à la question « Avez-vous totalement récupéré les capacités olfactives que vous aviez avant le début de l'apparition des symptômes de COVID-19 ? ») dans les deux groupes, le traitement par lavage de nez sera stoppé, mais il leur sera demandé de poursuivre la rééducation olfactive, deux fois par jour, jusqu'à la seconde visite de suivi.

Pour les patients du groupe expérimental n'ayant pas totalement récupéré, le traitement par budésonide en lavage de nez en complément de la rééducation olfactive sera poursuivi pendant 30 jours supplémentaires.

Pour les patients du groupe contrôle n'ayant pas totalement récupéré, le traitement par budésonide en lavage de nez sera instauré, en complément de la rééducation olfactive pendant 30 jours.

✓ ***<u>Visite de suivi n°3 (J60) :</u>***

Les patients seront revus à J60 (jusqu'à J70). Le test olfactif sera réalisé et la survenue d'événements indésirables sera recueillie dans l'e-CRF. Le questionnaire d'auto-évaluation des capacités olfactives et gustatives sera également réalisé (cf. **Annexe 2**).

A la suite de cette visite, la prise en charge des patients se fera selon les modalités habituelles du médecin suivant le patient.

✓ ***<u>Visite de suivi n°4 (M8) :</u>***

Une consultation téléphonique sera réalisée 8 mois (± 2 semaines) après l'inclusion des patients. Au cours de cette consultation sera réalisé le questionnaire d'auto-évaluation des capacités olfactives et gustatives (cf. **Annexe 2**).

La participation des patients prendra fin à l'issue de cette consultation téléphonique.

5.2.3 *Modalités de sorties d'étude*

✓ *<u>Arrêt de participation d'une personne à la recherche</u>*

Le patient pourra retirer son consentement et demander à sortir de l'étude à n'importe quel moment et quelle qu'en soit la raison. Les données recueillies au cours de sa participation à l'étude seront conservées à moins que le patient ne s'y oppose.

L'investigateur pourra interrompre temporairement ou définitivement la participation d'un patient à l'étude pour toute raison qui servirait au mieux les intérêts du patient en particulier en cas d'événements indésirables. La reprise de symptômes de COVID-19 mentionnés lors de l'appel téléphonique de suivi à J8 ou à n'importe quel autre moment de l'étude, conduiront à un arrêt du traitement mais au maintien de la surveillance liée à l'étude. Ils devront être rapportés sans délai au promoteur (cf 7.2.3).

En cas de sortie prématurée, l'investigateur devra en documenter les raisons de façon aussi complète que possible.

En cas de patient perdu de vue, l'investigateur mettra tout en œuvre pour reprendre contact avec la personne afin de connaître les raisons de sa sortie d'essai et son état de santé. Le cahier d'observation devra être rempli jusqu'à la dernière visite effectuée.

- ✓ *Modalités de suivi des patients sortis d'étude*

La sortie d'étude d'un patient ne changera en rien la prise en charge habituelle par rapport à sa maladie.

- ✓ *Participation à une autre recherche simultanément ou à la fin de celle-ci*

Les patients pourront participer à d'autres études si l'investigateur considère que celles-ci n'interfèrent pas avec la présente recherche.

Aucune période d'exclusion n'est prévue au-delà du suivi.

- ✓ *Arrêt définitif ou temporaire de la recherche*

L'étude pourra être suspendue ou interrompue en cas de survenue d'événements indésirables nécessitant une revue du profil d'innocuité du médicament étudié.

De même, des événements imprévus ou de nouvelles informations relatives au médicament étudié, au vu desquels les objectifs de l'étude ne seront vraisemblablement pas atteints, pourront amener le promoteur à interrompre prématurément l'étude.

En cas d'arrêt prématuré de l'étude, le promoteur informera l'ANSM et le CPP sans délai et au maximum dans un délai de 15 jours.

5.2.4 *Recueil des données*

Les données seront collectées dans un cahier d'observation électronique (e-CRF) via le logiciel Clinfile©, proposant un hébergement sécurisé des données, certifié ISO/IEC 27001. Toutes les données seront saisies dans l'e-CRF par l'équipe d'investigation de l'étude. Un numéro d'identification unique sera attribué à chaque patient, permettant la pseudonymisation des données. Pour chaque patient, les données directement identifiantes ne seront pas saisies dans l'e-CRF (i.e. seuls le numéro d'inclusion, la date d'inclusion, la première lettre du nom, la première lettre du prénom, le mois et l'année de naissance seront recueillis). Un fichier de correspondance permettant de faire le lien entre la base pseudonymisée et l'identité des patients sera conservé dans le classeur investigateur.

L'e-CRF sera créé, testé et validé avant le début de la saisie des données. Les données nécessaires à la réponse aux critères de jugement principal et secondaires seront monitorées à intervalles réguliers tout au long de l'essai. Les données seront monitorées à l'aide de règles prédéfinies de gestion des données et des requêtes seront automatiquement éditées. Tous les efforts raisonnables devront être faits pour compléter les données le plus tôt possible. L'investigateur principal est responsable de l'exactitude et de l'exhaustivité des données enregistrées dans le cahier d'observation. Après contrôle et validation, la base de données sera gelée et exportée pour analyse.

5.2.5 *Tableau récapitulatif du déroulement de l'étude*

Les examens notés S sont réalisés dans le cadre du soin et ceux notés R sont ajoutés du fait de la recherche.

	**Screening**	**Inclusion (J-1)**	***Appel téléphonique J8***	***Visite N°1 J15***	***Visite N°2 J30***	**Visite N°3 *J60***	**Visite N°4 M8**
**Mode de réalisation de la visite**	*Téléphone*	*Consultation*	*Téléphone*	*Téléphone*	*Consultation*	*Consultation*	*Téléphone*
Information	R						
Vérification des critères d'inclusion et de non inclusion	R	R					
Recueil du consentement écrit		R					
Test olfactif ODORATEST (Annexe 1)		S			S	S	
Questionnaire patient sur les capacités olfactives et gustatives (Annexe 2)		S			S	S	R
IRM 3T	S				R		
Recueil des événements indésirables		R	R	R	R	R	

5.3 Calendrier de l'étude

Nombre de patients attendus	120
Période d'inclusion	3 mois
Temps de participation maximum par patient	8 mois
Durée totale de l'étude	11 mois

5.4 Bénéfices, risques et contraintes pour le patient participant à la recherche

✓ *Bénéfices*

Cette étude a pour objectif de déterminer la meilleure prise en charge pour les patients présentant une perte de l'odorat suite à des symptômes de COVID-19. Les résultats nous permettront de formuler des recommandations concernant la prise en charge pour l'ensemble des patients souffrant d'une perte d'odorat persistante consécutive à une infection COVID-19. Par ailleurs, en termes de bénéfice individuel, nous nous attendons à une meilleure récupération des capacités olfactives chez les patients traités par budésonide.

✓ *Risques*

Ils procèdent des effets indésirables du budésonide. Compte tenu de la voie d'administration, les effets attendus sont surtout locaux et locorégionaux.
En se basant sur les effets du budésonide en suspension nasale, les effets indésirables potentiels sont :
-fréquent (≥1/100, <1/10) : épistaxis, sécrétions hémorragiques, irritation nasale, douleurs oropharyngées ;
- rare (≥ 1/10000, <1/1000) : dysphonie, vision floue, pression oculaire augmentée ;
-fréquence indéterminée : céphalées, candidoses nasales et oropharyngées, cataracte et glaucome auxquels s'ajoutent les réactions d'hypersensibilité peu fréquentes (≥ 1/1000, <1/100) : érythème, urticaire, rash, prurit, angiodème.
La survenue d'effets systémiques liés aux glucocorticoïdes est peu probable du fait de la voie d'administration, de la dose administrée et du temps d'exposition.

✓ *Contraintes*

Les contraintes liées au protocole sont considérées comme minimes. Elles relèvent de l'examen IRM réalisé après 30 jours de traitement, spécifiquement pour les besoins de la recherche. Les tests olfactifs font partie du soin courant. La durée de l'examen IRM sera d'environ 20 minutes.

Les patients pourront participer simultanément à une autre recherche, si le médecin investigateur considère qu'elle n'interfère pas avec cette étude.

Aucune indemnisation n'est prévue pour la participation des patients.

6 Description des médicaments de l’étude

6.1 Identification du traitement

- Dénomination commune internationale : Budésonide 1,00 mg pour un récipient unidose de 2 mL
- Forme et présentation : Suspension pour inhalation par nébuliseur
- Titulaire de l’AMM : TEVA Santé
- Date de l’AMM : 25/08/2009
- Composition :
    - Principe actif : budésonide
    - Excipients : edétate disodique, chlorure de sodium, polysorbate 80, acide citrique, citrate de sodium, eau pour préparations injectables

6.2 Conditionnement et étiquetage

Il s’agit d’une étude ouverte. Le conditionnement du médicament ne sera pas modifié. Le circuit et les numéros de lot devront être tracés. Un contre-étiquetage sera réalisé par la pharmacie de chaque centre afin d’identifier les traitements de l’étude. Le pharmacien s’engagera à ce que cet étiquetage n’occulte pas les mentions originales du produit commercial.

6.3 Commande et distribution des traitements

Les traitements seront commandés par la pharmacie responsable des essais cliniques de chaque centre. Le pharmacien/préparateur en pharmacie procédera au contre-étiquetage du conditionnement secondaire, conformément à la règlementation en vigueur.
Chaque centre investigateur pourra se fournir chez son fournisseur (même laboratoire pour le Budésonide et le NaCl 0,9 % 250 ml). La traçabilité des traitements expérimentaux sera assurée conformément à la règlementation des essais cliniques. Le circuit des traitements, les documents de traçabilité et les modalités de distribution et d’administration seront répertoriés dans les Procédures Circuit Pharmaceutique.

L’observance quotidienne des patients quant à la réalisation des lavages de nez et de la rééducation olfactive sera suivie à l’aide du **carnet patient**.

6.4 Administration

Le traitement sera administré localement en lavage de nez (3 seringues de 20cc dans chaque narine matin et soir). Les modalités de reconstitution des traitements et de réalisation des lavages de nez seront expliquées dans le **carnet patient** qui sera remis avec les traitements.

6.5 Adaptation de la posologie

Aucune adaptation de posologie n’est prévue. Le traitement pourra être interrompu transitoirement en cas de survenue d’effets indésirables.

6.6 Traitements associés, autorisés, non autorisés et de secours dans le cadre du protocole

Dans le groupe expérimental, il est déconseillé d'administrer conjointement un corticoïde, par voie inhalée ou orale ainsi qu'un inhibiteur puissant du CYP3A4 (bocéprevir, clarithromycine, cobicistat, érythomycine, itraconazole, kétoconazole, posaconazole, ritonavir, télithromycine, voriconazole).

Il n'existe aucun traitement de secours pour contrecarrer l'effet du médicament expérimental.

7 Evaluation de la sécurité

7.1 Définitions

Évènement indésirable : toute manifestation nocive survenant chez une personne qui se prête à une recherche impliquant la personne humaine que cette manifestation soit liée ou non à la recherche ou au produit sur lequel porte cette recherche.
Effet indésirable : toute réaction nocive et non désirée à un médicament expérimental quelle que soit la dose administrée.
Évènement (ou/effet) indésirable grave (EIG) : tout évènement (ou effet) indésirable qui :

- entraîne la mort,
- met en jeu le pronostic vital de la personne qui se prête à la recherche,
- nécessite une hospitalisation ou la prolongation d'une l'hospitalisation,
- provoque une incapacité ou un handicap important ou durable,
- se traduit par une anomalie ou une malformation congénitale (exposition intra utero),
- *ainsi que tout évènement (ou effet) considéré comme médicalement significatif (c'est à dire ayant des conséquences cliniques importantes mais ne correspondant pas à l'un des autres critères de gravité).*

et s'agissant du médicament, quelle que soit la dose administrée.

Effet indésirable inattendu : tout effet indésirable du produit dont la nature, la sévérité, la fréquence ou l'évolution ne concorde pas avec les informations de référence sur la sécurité mentionnées dans le résumé des caractéristiques du produit ou dans la brochure pour l'investigateur lorsque le produit n'est pas autorisé.
Lien de causalité : relation entre l'événement indésirable et le médicament (ou l'étude). Les facteurs à prendre en compte pour la détermination de l'imputabilité sont : les critères chronologiques (délai de survenue de l'effet indésirable, évolution de l'effet indésirable à l'arrêt du traitement, réapparition à la reprise), et les critères sémiologiques (mécanisme d'action ou explication pharmacodynamique, facteurs favorisants ou antécédents similaires, diagnostics différentiels possibles, examens complémentaires prouvant la cause).

- *non lié* : l'évènement est clairement lié à d'autres causes comme *non lié* : l'évènement est clairement lié à d'autres causes comme une progression de la maladie, un traitement concomitant, une maladie intermittente
- *possiblement lié* : évènement clinique ou biologique avec une relation chronologique et sémiologique compatible

Intensité : l'intensité des événements est évaluée par l'investigateur selon la classification suivante : Grade 1 : Léger ; asymptomatique ou symptômes légers ; diagnostic à l'examen clinique uniquement ; ne nécessitant pas de traitement - Grade 2 : Modéré ; nécessitant un traitement minimal, local ou non-invasif ; interférant avec les activités instrumentales de la vie quotidienne - Grade 3 : Sévère ou médicalement significatif mais sans mise en jeu immédiate du pronostic vital ; indication

d'hospitalisation ou de prolongation d'hospitalisation ; invalidant ; interférant avec les activités élémentaires de la vie quotidienne - Grade 4 : Mise en jeu du pronostic vital ; nécessitant une prise en charge en urgence - Grade 5 : Décès lié à l'EI

Incident : Fait susceptible d'affecter la qualité et la sécurité d'emploi du produit et donc de représenter un risque pour la santé des personnes. Cet incident peut survenir au cours de la chaîne de fabrication du produit de santé et jusqu'à son utilisation. Il est susceptible d'entraîner un effet indésirable.

Incident grave : Incident susceptible d'entraîner des effets indésirables graves.

Fait nouveau : Il s'agit de toute nouvelle donnée pouvant conduire à :

- ✓ une réévaluation du rapport des bénéfices et des risques de la recherche ou du produit objet de la recherche
- ✓ des modifications dans l'utilisation de ce produit, dans la conduite de la recherche, ou des documents relatifs à la recherche
- ✓ ou à suspendre ou interrompre ou modifier le protocole de la recherche ou des recherches similaires. Pour les essais portant sur la première administration ou utilisation d'un produit de santé chez des personnes qui ne présentent aucune affection : tout effet indésirable grave.

Un fait nouveau peut également correspondre à une suspicion d'effet indésirable grave inattendu *(SUSAR*).

Pour les essais portant sur la première administration ou utilisation d'un produit de santé chez des personnes qui ne présentent aucune affection : tout effet indésirable grave est considéré comme un fait nouveau.

7.2 Enregistrement et notification des évènements indésirables par l'investigateur

7.2.1 *Evènements indésirables*

L'investigateur reporte les évènements indésirables dans le cahier d'observation.

Il évalue pour chaque évènement indésirable, son intensité, sa gravité et le lien de causalité avec la procédure/la technique étudiée (ou comparative) ou avec les autres traitements éventuels.

Les évènements indésirables couvrent également les erreurs médicamenteuses et les utilisations non prévues par le protocole y compris les mésusages ou l'abus de médicament.

7.2.2 *Evènements indésirables graves*

L'investigateur notifie **sans délai dès qu'il en a connaissance** les événements indésirables graves (EIG) au promoteur, à l'aide du « formulaire de notification d'EIG ».

L'évolution clinique ainsi que les résultats des éventuels bilans cliniques et des examens diagnostiques et/ou de laboratoire, ou toute autre information permettant une analyse adéquate du lien de causalité de l'EIG sont à préciser :

- soit sur la notification initiale d'EIG s'ils sont immédiatement disponibles,
- soit ultérieurement et le plus rapidement possible, sur un nouveau « formulaire de notification d'EIG » de suivi (le 1er suivi à adresser dans les 8 jours après la notification initiale).

La notification initiale, et, le cas échéant, ses rapports de suivi, sont transmis par email ou par télécopie au promoteur :

Vigilance des Essais Cliniques, Service de Recherche Clinique
Fondation A. de Rothschild (Paris)
Mail : shoudas@for.paris
Télécopie : 01 48 03 64 30

L'investigateur communique au promoteur tous les renseignements complémentaires demandés (compte-rendu d'examens complémentaires, compte-rendu d'hospitalisation, résultats d'autopsie, etc.).
Chaque document transmis par l'investigateur devra comporter l'acronyme de la recherche et l'identifiant du patient attribué dans le cadre de la recherche. L'original des documents transmis est conservé sur le site d'investigation.
Tout patient présentant un EIG recevra la prise en charge adaptée à son état et sera suivi jusqu'à la résolution de l'événement ou à un retour à l'état antérieur. Si cela s'avère nécessaire, le médicament expérimental sera arrêté.

Tout EIG doit être notifié au promoteur :

- à partir de la date de signature du consentement,
- jusqu'à la fin de participation à la recherche,

mais également sans limitation de temps, lorsque l'EIG est susceptible d'être dû au médicament.

7.2.3 *Evènements indésirables d'intérêt spécifique*

L'investigateur notifie sans délai dès qu'il en a connaissance au promoteur toute suspicion de réapparition de symptômes d'infection à COVID-19 (fièvre, toux, gêne thoracique, dyspnée, asthénie …), à l'aide du « formulaire de notification d'EIG » car ils nécessitant une vigilance particulière *(s'ils ne répondent pas à la définition des EIG, ils seront classés comme « médicalement significatifs).*

7.2.4 *Evènement indésirables graves ne nécessitant pas de notification immédiate*

Les évènements indésirables graves ne devant pas être notifiés immédiatement au promoteur sont :

- Une hospitalisation pour une procédure médicale/chirurgicale prévue avant l'inclusion ou prévue dans le protocole ;
- Une hospitalisation pour une pathologie présente avant l'inclusion et ne s'aggravant pas en cours d'étude ;
- Une circonstance de vie n'ayant aucune incidence sur l'état de santé et ne nécessitant aucune intervention médicale/chirurgicale (ex : prolongation d'hospitalisation en attendant une place dans un autre service ou établissement, hospitalisation pour cause d'aidants indisponibles, etc…).

7.3 Prise en charge des évènements indésirables graves par le promoteur

7.3.1 *Evaluation médicale*

Pour chaque EIG, le promoteur (le vigilant) évalue de son côté la gravité et le lien avec le médicament, il juge du caractère attendu (listé) ou inattendu selon le document de référence.
Il sollicite l'investigateur (ou l'ARC/TEC de l'étude), pour obtenir les informations complémentaires sur les circonstances de survenue de l'EIG, son traitement et son évolution.
Il code l'EIG selon la méthode MedDRA et rédige le narratif de l'EIG en anglais

7.3.2 *Déclaration des suspicions d'effets indésirables graves inattendus (SUSAR)*

Le promoteur déclare à l'ANSM toute suspicion d'effet indésirable grave inattendu (SUSAR : *suspected unexpected serious adverse reaction*) :

- En cas de décès ou de mise en jeu du pronostic vital : sans délai à compter du jour où il en a connaissance
- Dans les autres cas ; au plus tard dans un délai de 15 jours à compter du jour où il en a connaissance

Les informations complémentaires pertinentes doivent être adressées dans les 8 jours.

Le promoteur informe les investigateurs de l'étude des suspicions d'effets indésirables graves inattendus qui pourraient avoir un impact défavorable sur la sécurité des personnes qui se prêtent à la recherche.

7.4 Fait nouveau et mesures urgentes de sécurité

En cas de survenue d'un fait nouveau durant l'étude, le promoteur en informe sans délai, l'ANSM et le CPP en précisant les éventuelles mesures de sécurité prises
Les informations complémentaires pertinentes doivent être adressées dans les 8 jours.

De plus, en cas :

- D'arrêt de la recherche : le promoteur déclare la fin de la recherche sans délai et au plus tard dans les 15 jours.
- De modification substantielle : le promoteur dépose une demande de modification substantielle dans un délai de 15 jours.

7.5 Rapport annuel de sécurité

Une fois par an pendant toute la durée de la recherche, le promoteur rédige un rapport de sécurité. Ce rapport de sécurité comprend notamment une analyse globale du profil de sécurité du protocole de l'étude prenant en compte toutes les nouvelles données pertinentes de sécurité.

Les informations de sécurité apparaissent sous forme de tableaux de synthèse résumant les évènements ou effets indésirables graves survenus au cours de la recherche.
La date de début du rapport est la date d'autorisation initiale de l'ANSM. Ce rapport est envoyé électroniquement à l'ANSM et au CPP dans un délai de 60 jours suivant la date anniversaire de l'autorisation par l'ANSM.

Dans le cas de cette recherche prévue sur quelques mois, le promoteur rédigera un rapport de sécurité ayant comme date de fin, le dernier suivi du dernier patient inclus.

7.6 Comité de surveillance indépendant

Un comité de surveillance indépendant sera mis en place pour cette étude. Il inclura un méthodologiste, un ORL et un infectiologue, tous trois indépendants de l'étude et n'exerçant pas au sein des centres investigateurs. Une charte de fonctionnement a été rédigée concernant le fonctionnement de ce comité. Les comptes rendus de réunions du CSI seront fournis à l'ANSM dès que disponibles.

8 Analyses statistiques

8.1 Calcul du nombre de sujets

Nous faisons l'hypothèse que le pourcentage de patients présentant une amélioration olfactive dans le groupe contrôle sera d'environ 50% après 30 jours.
Afin de mettre en évidence une différence de 25% entre les deux bras (50% versus 75% de patients présentant une amélioration olfactive dans le bras traité par budésonide), environ 60 patients par bras sont nécessaires, **soit un total de 120 participants** (formulation bilatérale, puissance=0,8, alpha=0,05).

8.2 Description des analyses statistiques utilisées

L'analyse statistique sera réalisée sous la responsabilité du service de recherche clinique de la Fondation Adolphe de Rothschild.

8.2.1 *Choix des personnes à inclure dans les analyses*

L'ensemble des patients randomisés ayant reçu au moins une dose de traitement seront pris en compte dans les analyses.

8.2.2 *Statistiques descriptives*

Un diagramme de flux établi selon les recommandations CONSORT résumera le nombre de patients screenés, inclus, randomisés, perdus de vue et analysés pour chaque bras de randomisation. Les raisons de non-participation à chaque étape seront décrites.

Une analyse descriptive des données sera réalisée. Cette analyse comportera des estimations ponctuelles, nombres et pourcentages pour les variables qualitatives, moyenne, écart-type, médiane et range pour les variables quantitatives. La normalité des variables continues sera évaluée graphiquement et/ou à l'aide d'un test de normalité (e.g. Shapiro-Wilk). Une description des données manquantes de chaque variable (effectif et pourcentage) sera réalisée.

8.2.3 *Comparaison des groupes à la baseline*

La comparabilité à la baseline entre le bras expérimental et le bras contrôle sera évaluée au moyen d'un test t de Student (ou test de Mann-Whitney si nécessaire) pour les paramètres continus et d'un test de Chi^2 (ou test exact de Fisher si nécessaire) pour les paramètres qualitatifs.

8.2.4 *Analyse du critère de jugement principal*

L'analyse du critère de jugement principal sera réalisée à l'aide d'un modèle de régression logistique, ajusté sur le centre.

8.2.5 *Analyse des critères de jugement secondaires*

Les objectif et critères de jugement secondaires de l'étude sont :

1- Objectif : Chez les patients ayant totalement récupéré après 30 jours de traitement, évaluer la persistance de la récupération olfactive 1 mois après l'arrêt du traitement.

Critère : Pourcentage de patients ayant une amélioration de plus de 2 points au score ODORATEST 30 jours après la fin du traitement (i.e. 60 jours après la randomisation).

2- Objectif : Dans le groupe traité par budésonide, chez les patients COVID-19 n'ayant pas totalement récupéré après 30 jours de traitement, évaluer l'intérêt d'une poursuite du budésonide pendant 30 jours supplémentaires.

Critère : Pourcentage de patients ayant une amélioration de plus de 2 points au score ODORATEST après 60 jours de traitement par budésonide.

3- Objectif : Dans le groupe contrôle, chez les patients COVID-19 n'ayant pas totalement récupéré après 30 jours de traitement, évaluer l'intérêt de la mise en place d'un traitement par budésonide pendant 30 jours.

Critère : Pourcentage de patients ayant une amélioration de plus de 2 points au score ODORATEST 30 jours après l'initiation d'un traitement par budésonide (i.e. 60 jours après la randomisation).

4- Objectif : Rechercher une association entre la présence d'une obstruction à l'IRM et la sévérité de la perte olfactive, lors de l'inclusion et après 30 jours de traitement.

Critère : Présence d'un comblement inflammatoire de la fente olfactive à l'IRM ou présence d'une atteinte neurologique au niveau du bulbe olfactif.

5- Objectif : Evaluer la tolérance du budénoside.

Critère : Description des évènements indésirables et événements indésirables graves.

6- Décrire les capacités olfactives et gustatives des patients à l'aide d'un questionnaire d'auto-évaluation.

Critère : Questionnaire d'auto-évaluation (cf. **Annexe 2**).

7- Objectif : Comparer au bout de 30 jours de traitement les capacités olfactives entre les deux bras

Critère : Score ODORATEST après 30 jours de traitement

8- Comparer au bout de 30 jours de traitement les capacités olfactives de type « détection des odeurs » entre les deux bras

Critère : sous-score détection de l'ODORATEST après 30 jours de traitement

9- Comparer au bout de 30 jours de traitement les capacités olfactives de type « identification des odeurs » entre les deux bras

Critère : sous-score identification de l'ODORATEST après 30 jours de traitement

Les critères 1, 2, 3, et 5 et 6 seront décrits selon les modalités détaillées dans la section 8.2.2.

Le critère 4 sera analysé à l'aide d'un test du Chi2 (ou test exact de Fisher si nécessaire).

Les critères 7, 8 et 9 seront analysés à l'aide de tests de Student (ou tests de Wilcoxon si nécessaire)

8.3 Niveau de significativité statistique

L'ensemble des tests seront réalisés selon une formulation bilatérale. Une valeur de $p < 0.05$ sera considérée comme statistiquement significative.

8.4 Critères statistiques d'arrêt de la recherche

Non applicable.

8.5 Modalités de prise en compte des données manquantes, non utilisées ou non valides

Pour l'analyse du critère de jugement principal, en cas de données manquantes, une imputation sera réalisée selon la méthode BOCF (Baseline Observation Carried Forward) : les patients non évalués à J30 seront considérés comme n'ayant pas présenté d'amélioration des capacités olfactives.

8.6 Gestion des modifications apportées au plan statistique initial

Des modifications à la stratégie initiale pourront être apportées, en fonction notamment de l'avancement de l'étude, et reprises dans le plan d'analyse statistique qui sera rédigé juste avant le gel de la base de données. Les éventuelles modifications à apporter au plan d'analyse statistique seront proposées par le méthodologiste de l'étude et validées par consensus avec le statisticien et l'investigateur principal.

9 Dispositions réglementaires

La Fondation Adolphe de Rothschild est promoteur de cette **recherche impliquant la personne humaine, de type interventionnelle**, selon l'article L1121-1 1er alinéa du Code de la Santé Publique, modifié par Ordonnance n°2016-800 du 16 juin 2016 (art. 1).

9.1 Demande d'autorisation auprès de l'autorité compétente (Agence Nationale de Sécurité du Médicament et des produits de santé - ANSM)

Le présent protocole fera l'objet d'une demande d'autorisation auprès de l'autorité compétente (ANSM). L'autorité compétente, définie à l'article L. 1123-12, modifié par Ordonnance n°2016-800 du 16 juin 2016, se prononce au regard de la sécurité des personnes qui se prêtent à une recherche interventionnelle, en considérant notamment la sûreté et la qualité des produits utilisés au cours de la recherche conformément, le cas échéant, aux référentiels en vigueur, leur condition d'utilisation et la sécurité des personnes au regard des actes pratiqués et des méthodes utilisées ainsi que les modalités prévues pour le suivi des personnes. Elle se prononce en outre sur la pertinence de la recherche, le caractère satisfaisant de l'évaluation des bénéfices et risques attendus et le bien-fondé des conclusions.

9.2 Demande d'avis auprès du Comité de Protection des Personnes (CPP)

En accord avec l'article L.1123-6 du Code de Santé Publique modifié par Ordonnance n°2016-800 du 16 juin 2016 (art 3.), cette recherche impliquant la personne humaine est soumise à l'avis d'un CPP désigné de manière aléatoire, sans lequel elle ne pourra débuter. L'avis de ce comité sera notifié à l'autorité compétente par le promoteur avant le démarrage de la recherche.

9.3 Engagements – aspects éthiques

Le promoteur et les investigateurs s'engagent à ce que cette recherche soit réalisée en conformité avec les dispositions législatives en vigueur, les Bonnes Pratiques Cliniques et la déclaration d'Helsinki.

Les investigateurs s'engagent à respecter le protocole en tout point en particulier en ce qui concerne le recueil du consentement et la notification et le suivi des événements indésirables graves. Un exemplaire

de l’engagement de responsabilités daté et signé par les investigateurs sera remis au représentant du promoteur.

9.4 Déclaration du démarrage et de la fin de l’étude

Dès la première inclusion, le promoteur informera sans délai l’ANSM et le CPP de la date effective de démarrage de l’étude. La date effective de démarrage correspond à la date de signature du consentement par la première personne qui se prête à la recherche.
La date de fin d’étude sera transmise par le promoteur à l’ANSM et au CPP dans un délai de 90 jours. La date de fin de la recherche correspond au terme de la participation de la dernière personne qui se prête à la recherche, ou le cas échéant, au terme défini dans le protocole.

9.5 Note d’information et recueil du consentement écrit du patient

Une note d’information précisant les informations à fournir selon l’article 13 du Règlement Général sur la Protection des Données de l'Union européenne (RGPD) et soumise préalablement au CPP, sera expliquée et remise aux patients qui peuvent refuser de participer à la recherche, comme précisé dans le document. Le détail de la procédure d’information est mentionné plus haut dans ce protocole.
L’information donnée aux personnes et le recueil de leur consentement doivent être notés et datés dans le dossier médical du patient. En cas de refus de participation, le patient bénéficiera de la prise en charge habituelle. Lorsque cette recherche sera terminée, la personne qui se prête à la recherche pourra être informée des résultats globaux selon les modalités qui lui seront précisées dans le document d'information. Les informations relatives aux droits des personnes participant à cette recherche (droit d’accès et de rectification, droit d’opposition à la transmission des données couvertes par le secret professionnel susceptibles d’être utilisées dans le cadre de cette recherche) sont intégrées dans la note d’information.

9.6 Modifications du protocole

L’investigateur coordonnateur informera le promoteur de tout projet de modification du protocole.

Le promoteur est seul autorisé à modifier le protocole et décidera si la modification envisagée relève ou non d’une modification substantielle. La demande de modification sera adressée par le promoteur à l’ANSM et/ou au CPP pour autorisation et avis selon les cas.

On entend par modifications substantielle : les modifications qui ont un impact significatif sur tout aspect de la recherche, notamment sur la protection des personnes, y compris à l’égard de leur sécurité, sur les conditions de validité de la recherche, le cas échéant sur la qualité et la sécurité des produits expérimentés, sur l’interprétation des documents scientifiques qui viennent appuyer le déroulement de la recherche ou sur les modalités de conduite de celle-ci. Une modification non substantielle du protocole est une modification mineure ou une clarification sans retentissement sur la conduite de l’essai.

Dès réception de l’avis et/ou de l’autorisation, la version amendée du protocole sera alors transmise à tous les investigateurs par le promoteur.

9.7 Déclaration CNIL (Commission Nationale de l’Informatique et des Libertés)

Cette recherche est soumise à la loi n°78-17 du 6 janvier 1978 relative à l’informatique, aux fichiers et aux libertés modifié par la loi n°2018-493 du 20 juin 2018 relative à la protection des données personnelles, ainsi qu’au Règlement Général sur la Protection des Données de l'Union européenne (RGPD). Dans le cadre du traitement de données de santé à des fins de recherche scientifique, cette recherche est soumise à l’article 9 du RGPD, alinéas I et J de l’article 9.2.

Une méthodologie de référence spécifique au traitement de données personnelles opéré dans le cadre des recherches impliquant la personne humaine, de type interventionnelle, a été établie par la CNIL (MR-001). Cette méthodologie permet une procédure de déclaration simplifiée lorsque la nature des données recueillies dans la recherche et leur traitement sont compatibles avec le document de référence de la CNIL. Les données recueillies au cours de l'étude ne mentionneront ni le nom ni le prénom des patients inclus, mais seront codées. Les renseignements obtenus seront sauvegardés sur fichier informatique. Seules les personnes directement impliquées dans l'étude seront habilitées à intervenir sur ces fichiers. Les informations relatives aux droits des personnes participant à cette recherche (droit d'accès et de rectification, droit d'opposition à la transmission des données couvertes par le secret professionnel susceptibles d'être utilisées dans le cadre de cette recherche) seront intégrées dans les notes d'information.

9.8 Droits d'accès aux données et documents sources

Toutes les données et informations concernant le patient resteront strictement confidentielles. Les personnes ayant un accès direct conformément aux dispositions législatives et réglementaires en vigueur, notamment les articles L.1121-3 et R.5121-13 du Code de la Santé Publique (par exemple, les investigateurs, les personnes chargées du contrôle de qualité, les moniteurs, les assistants de recherche clinique, les auditeurs et toutes personnes appelées à collaborer aux essais) prendront toutes les précautions nécessaires en vue d'assurer la confidentialité des informations relatives au médicament expérimental, aux essais, aux personnes qui s'y prêtent et notamment en ce qui concerne leur identité ainsi qu'aux résultats obtenus. Les données collectées par ces personnes au cours des contrôles de qualité ou des audits seront alors rendues anonymes.

L'investigateur garantira l'accès aux données sources pour le moniteur, l'auditeur ou l'inspecteur de l'autorité administrative compétente. Il s'engage à accepter les contrôles du promoteur et à fournir l'accès aux données sources (dossiers médicaux, fichiers informatiques, documents de l'étude,…).

Des visites de monitoring seront effectuées dans chaque centre selon une fréquence définie par le promoteur. Lors de ces visites sur site en accord avec l'investigateur, les éléments suivants sont revus :
- respect du protocole et des procédures qui y sont rattachées,
- assurance qualité des données recueillies dans le cahier d'observation : exactitude, données manquantes, cohérence des données, contrôle des documents sources.

Les participants ont un droit de limitation de traitement de leurs données personnelles selon les conditions décrites à l'article 18 du RGPD.

Les données pourront être consultées uniquement par le personnel autorisé dans le cadre d'un contrôle qualité.

9.9 Archivage

Les documents et données relatifs à cette recherche seront archivés par l'investigateur et par le promoteur, pour une durée de 15 ans après la fin de la recherche.
Cet archivage indexé comporte :
- Les copies de l'autorisation de l'ANSM
- Les copies de l'avis du CPP
- Les versions successives du protocole (identifiées par le n° de version et la date de version)
- Les courriers de correspondance entre promoteur et les investigateurs
- Le cahier d'observation complété et validé de chaque sujet inclus

- Les formulaires de consentement
- Toutes les annexes spécifiques à l’étude (notamment le CV des investigateurs)
- Le rapport final de l’étude provenant de l’analyse statistique et du contrôle qualité de l’étude (double transmis au promoteur)
- Les certificats d’audits éventuels réalisés au cours de la recherche
- La base de données ayant donné lieu à l’analyse statistique, devant aussi faire l’objet d’archivage par le responsable de l’analyse (support papier ou informatique)

10 Assurance et financement

10.1 Assurance

Dans cette recherche impliquant la personne humaine, de type interventionnelle, la Fondation Adolphe de Rothschild, en tant que promoteur, contractera auprès de la SHAM une police d’assurance pour toute la durée de la recherche. Cette assurance garantira sa propre responsabilité civile ainsi que celle de tout intervenant (médecin ou personnel impliqué dans la réalisation de la recherche), indépendamment de la nature des liens existant entre les intervenants et le promoteur (Article L1121-10 du Code de la Santé Publique, modifié par loi n°2012-300 du 5 mars 2012 – art. 1 et article R 1121-4, modifié par décret n°2106-1537 du 16 novembre 2016 – art.3).

10.2 Financement

Le promoteur prendra en charge les frais supplémentaires liés aux fournitures ou examens spécifiquement requis par le protocole de la recherche pour la mise en œuvre de celui-ci. Lorsque la recherche est réalisée dans un établissement de santé, la prise en charge de ces frais fait l'objet d'une convention entre le promoteur et le représentant légal de cet établissement.

11 Règles relatives à la publication et rapport final

11.1 Enregistrement de l’étude

L’étude sera enregistrée sur un site web en libre accès (e.g. https://clinicaltrials.gov/) avant l'inclusion du premier patient.

11.2 Rapport final

Le rapport d'étude final mentionné à l'article R.1123-67 du Code de la Santé Publique sera rédigé et signé par le promoteur et l'investigateur. Les résultats de l’étude seront transmis dans la base EudraCT dans un délai d'un an suivant la fin de l’essai, c’est-à-dire au terme de la participation de la dernière personne qui se prête à la recherche.

11.3 Communication et publication des résultats

Conformément à l’article R 5121-13 du Code de la Santé Publique, les essais ne peuvent faire l’objet d’aucun commentaire écrit ou oral sans l’accord conjoint de l’investigateur et du promoteur.

La Fondation Adolphe de Rothschild, promoteur de l’étude, est propriétaire des données et des résultats de l’étude. Aucune utilisation ou transmission à un tiers ne peut être effectuée sans son accord préalable.

Les communications et publications des résultats de l’étude seront réalisées sous la responsabilité de l’investigateur coordonnateur avec l’accord des investigateurs et des autres coauteurs associés. Elles

devront décrire de façon honnête et équilibrée tous les aspects de l'étude sans tenir compte d'autres intérêts, notamment non scientifiques. Les coauteurs des publications seront les investigateurs et les cliniciens impliqués (au prorata de leur contribution), le méthodologiste et le biostatisticien référent de l'étude, et les chercheurs associés (à définir selon le cas). Les autres membres du personnel du service de recherche clinique ayant activement participé à l'élaboration, au déroulement du protocole et à la rédaction des résultats devront également être cités. Les règles de publications suivront les recommandations du réseau EQUATOR (https://www.equator-network.org/reporting-guidelines/). La Fondation Adolphe de Rothschild sera mentionnée comme étant le promoteur de la recherche. Cette dernière a la maîtrise de la première publication.

Toute requête pour cacher des résultats, changer et atténuer le contenu du rapport final ou des publications sera systématiquement rejetée par le promoteur. L'investigateur adressera une copie des publications au promoteur.

12 Références

1. *Early Clinical and CT Manifestations of Coronavirus Disease 2019 (COVID-19) Pneumonia.* **Han, R, et al., et al.** 17, 2020, AJR Am J Roentgenol, pp. 1-6.
2. *Sixty seconds on . . . anosmia.* **Iacobucci, G.** 24 Mar 2020, BMJ , p. 1202.
3. *Olfactory neuropathy in severe acute respiratory syndrome: report of A case.* **Hwang, CS.** 1, Mar 2006, Acta Neurol Taiwan, Vol. 15, pp. 26-8.
4. *The relationship of olfactory function and clinical traits in major depressive disorder.* **Wang F, Wu X, Gao J, Li Y, Zhu Y, Fang Y.** 16, Mar 2020, Behav Brain Res , p. 112594.
5. **Tan, W, et al., et al.** Viral Kinetics and Antibody Responses in Patients with COVID-19. *medRxiv preprint.* [En ligne] 24 mar 2020. [Citation : 2 Apr 2020.]
6. *Clinical course and risk factors for mortality of adult inpatients with COVID-19 in Wuhan, China: a retrospective cohort study.* **Zhou, F, et al., et al.** 10229, 28 Mar 2020, Lancet, Vol. 395, pp. 1054-1062.
7. **Yan, CH, Overdevest, JB et Patel, ZM.** Therapeutic use of steroids in non-chronic rhinosinusitis olfactory dysfunction: a systematic evidence-based review with recommendations. *Int Forum Allergy Rhinol.* Feb 2019 , Vol. 9, 2, pp. 165-176.
8. **Nguyen, TP et Patel, ZM.** Budesonide irrigation with olfactory training improves outcomes compared with olfactory training alone in patients with olfactory loss. *Int Forum Allergy Rhinol.* Sep 2018, Vol. 8, 9, pp. 977-981.
9. *Olfactory dysfunction and its measurement in the clinic.* **Doty, RL.** 1, 26 Oct 2015, World J Otorhinolaryngol Head Neck Surg, Vol. 1, pp. 28-33.
10. *A new clinical olfactory test to quantify olfactory deficiences.* **Eloit, C et Trotier, D.** 1994, Rhinology.
11. **Gurgel R, Jackler R, Dobie R, Popelka G.** A new standardized format for reporting hearing outcome in clinical trials. *Otolaryngol Head Neck Surg.* 2012, Vol. 147, 5, pp. 803–7.

13 Annexes

13.1 Annexe 1. Test olfactif ODORATEST

L'olfactomètre utilisé pour cette étude permet de déterminer le seuil olfactif pour cinq odorants purs, facilement reconnaissables tels le PHENYLETHYL ALCOOL (odeur 1) évoquant l'odeur de rose ou fleur; le GAMMA1 DECALACTONE (odeur 2) : caramel ou gâteau ; l' ACIDE ISOVALERIQUE (odeur 3) : sueur ou fromage de chèvre ; le CYCLOTENE (odeur 4): abricot ou pêche et le SCATOLE (odeur 5): étable ou purin. Trois de ces odeurs sont dites « agréables » et deux sont dites « désagréables ». Toutes sont des odeurs familières.

Chaque substance est présentée à 8 niveaux de concentrations selon une échelle logarithmique (sauf pour les odeurs 2 et 5 pour lesquelles la concentration pure est impossible techniquement à obtenir ; pour ces deux odeurs, il existe donc 7 dilutions), chaque niveau différant du suivant d'un facteur 10 couvrant toute l'étendue de l'acuité olfactive humaine.

L'ensemble du test dure environ une heure. Il est géré par ordinateur pour une présentation aléatoire des stimuli et l'acquisition des données.

Le test est réalisé par le patient seul sans intervention d'une infirmière.

La première épreuve consiste en un apprentissage des odeurs et de la manipulation des flacons et du clavier.

La première série est présentée pour un ordre aléatoire en concentrations décroissantes, et il s'agit encore d'un exercice de familiarisation.

La deuxième série est présentée en ordre aléatoire et en concentrations croissantes. Il s'agit du test a proprement parler. Les réponses données par le patient seront enregistrées et permettront de donner un seuil de détection et d'identification pour chaque odeur. Le résultat du test est calculé en établissant la moyenne géométrique entre la dernière concentration non perçue et la première concentration perçue pour chaque produit. Le score final est la moyenne des seuils pour les cinq produits en détection et en identification.

Pour les odeurs 2 et 5, il est techniquement impossible d'avoir une concentration pure. Le score moyen le plus élevé est donc de 8,1 ce qui correspond à une anosmie totale.

13.2 Annexe 2. Questionnaire d'auto-évaluation

L'objectif de ce questionnaire est d'évaluer comment les gens perçoivent des changements dans leur sens du goût et de l'odorat. Merci de répondre aux questions suivantes du mieux possible.

1. Sur une échelle allant de 0 (pas de goût) à 10 (goût normal), à combien évaluez-vous votre goût aujourd'hui /10

2. J'ai un mauvais goût persistant dans la bouche (entourez une seule réponse)

    1. Jamais
    2. Rarement
    3. Parfois
    4. Souvent
    5. Toujours

3. Ce goût persistant est (entourez TOUTES les réponses qui conviennent)

    1. Salé
    2. Sucré
    3. Aigre (comme du citron ou du vinaigre)
    4. Amer (comme du café noir ou du tonic)
    5. Autre (précisez) : ………………………………..

4. Si je compare mon sens du goût maintenant relativement à avant de tomber malade :

    • Je perçois le goût salé (entourez une seule réponse)

        1. Plus fort
        2. Aussi fort
        3. Moins fort
        4. Je ne le sens pas du tout

    • Je perçois le goût sucré (entourez une seule réponse)

        1. Plus fort
        2. Aussi fort
        3. Moins fort
        4. Je ne le sens pas du tout

    • Je perçois le goût aigre (citron ou vinaigre) (entourez une seule réponse)

        1. Plus fort
        2. Aussi fort
        3. Moins fort
        4. Je ne le sens pas du tout

• Je perçois le goût amer (café noir ou tonic) (entourez une seule réponse)

1. Plus fort
2. Aussi fort
3. Moins fort
4. Je ne le sens pas du tout

5. Je qualifierais cette anomalie de mon sens du goût comme (entourez une seule réponse)

1. Insignifiante
2. Légère
3. Modérée
4. Sévère
5. Invalidante

6. Cette perturbation du sens du goût affecte ma qualité de vie de manière (entourez une seule réponse)

1. Insignifiante
2. Légère
3. Modérée
4. Sévère
5. Invalidante

8. Sur une échelle allant de 0 (pas d'odorat) à 10 (odorat normal), à combien évaluez-vous votre odorat aujourd'hui /10

9. Si je compare mon sens de l'odorat maintenant relativement à avant de tomber malade, les odeurs sont (entourez une seule réponse)

1. Plus fortes
2. Aussi fortes
3. Moins fortes
4. Je ne sens rien du tout

10. Je qualifierais cette anomalie de mon sens de l'odorat comme (entourez une seule réponse)

1. Insignifiante
2. Légère
3. Modérée
4. Sévère
5. Invalidante

11. Cette perturbation du sens de l'odorat affecte ma qualité de vie de manière (entourez une seule réponse)

1. Insignifiante
2. Légère
3. Modérée

4. Sévère

5. Invalidante

13.3 Annexe 3 : Protocole de rééducation olfactive

La rééducation olfactive utilise des odorants aromatiques, faciles à se procurer (même en période de confinement). Elle est composée de 6 odorants : Vanille, clou de girofle, vinaigre, curry, cannelle et thym.

Les exercices sont à réaliser **le matin et le soir** (temps estimé 5 minutes) :

- Le matin « **à jeun** », avant de prendre le petit déjeuner (y compris boisson chaude) ;

- Le soir avant le dîner, avant même l'apéritif ;

- En évitant, de manière générale les perturbations olfactives (loin des repas ou du brossage des dents par exemple).

Les flacons sont identifiables (nom du produit visible) :

- Ouvrir le flacon en regardant le nom de l'odeur ;
- Renifler le contenu du flacon pendant 15 secondes;
- Placer chaque flacon à environ 2 cm du nez (pas trop près) et réalisant un balayage de droite à gauche.
- Attendre 15 secondes avant de passer au flacon suivant.
- Renouveler l'opération pour les 5 autres flacons.
